# An assessment of irrigated rice cultivation with different crop establishment practices in Vietnam

**DOI:** 10.1038/s41598-021-04362-w

**Published:** 2022-01-10

**Authors:** Van-Hung Nguyen, Alexander M. Stuart, Thi-My-Phung Nguyen, Thi-Minh-Hieu Pham, Ngoc-Phuong-Thanh Nguyen, Anny Ruth P. Pame, Bjoern Ole Sander, Martin Gummert, Grant Robert Singleton

**Affiliations:** 1 International Rice Research Institute, 4031 Los Baños, Laguna Philippines; 2 Department of Agricultural and Rural Development of Can-Tho City, Can-Tho City, Vietnam; 3 Institute of Sanitary Engineering and Waste Management, Leibniz Universität Hannover, Hannover, Germany; 4 Department of Biological Sciences, Northern Arizona University, Flagstaff, AZ 86001 USA

**Keywords:** Environmental impact, Sustainability, Climate-change mitigation

## Abstract

Overuse of seed and chemical inputs is a major constraint for sustainable rice production in Vietnam. In this study, two seasons of field trials were conducted to compare different crop establishment practices for rice production in the Mekong River Delta using environmental and economic sustainability performance indicators. The indicators including energy efficiency, agronomic use efficiency, net income, and greenhouse gas emissions (GHGEs) were quantified based on four treatments including manual broadcast-seeding, blower seeding, drum seeding, and mechanized transplanting. Across the four treatments, yields ranged from 7.3–7.5 Mg ha^−1^ and 6.2–6.8 Mg ha^−1^ in the Winter-Spring (WS) and Summer-Autumn (SA) seasons, respectively. In comparison with direct seeding methods, mechanized transplanting decreased the seed rate by 40%. It also led to a 30–40% reduction in pesticide use during the main crop season (WS). Mechanized transplanting required higher inputs, including machine depreciation and fuel consumption, but its net energy balance, net income and GHGE were at a similar level as the other non-mechanized planting practices. Mechanized transplanting is a technology package that should be promoted to improve the economic and environmental sustainability of lowland rice cultivation in the Mekong River Delta of Vietnam.

## Introduction

Rice is the main crop and major staple food of many Asian countries with global production at about 505 million tons annually^1^. Demand for rice is estimated to double when the global population reaches 9 billion by 2050^2^. However, rice production currently faces many serious challenges, such as climate change, labor shortage, water shortages and loss of crop lands because of increased urbanization and industrialization^3,4^. Flooded rice production has a substantial environmental footprint, such as contributing 1.5% to global GHGEs^5^. These challenges and problems of rice production are applicable in Vietnam, which is one of the top rice-exporting countries. Vietnam produces 6% of the global rice supply^1^. In addition, there are concerns about the effects of intensification of cereal cropping on biodiversity^6^. The current overuse of chemical inputs in rice production in Vietnam, such as fertilizers and pesticides, has adverse effects on biodiversity, the environment and human health^7–9^.

In Southeast Asian countries, most rice fields are fragmented with small plot sizes of about 0.1–2 ha, causing low energy efficiency and productivity^10,11^. In response to these challenges and problems, sustainable practices and programs are being promoted, such as Global G.A.P, VietGAP and the Sustainable Rice Platform (SRP) standards^12,13^. In the Mekong River Delta (MRD) of Vietnam, rice farmers have been adopting a set of best management practices named “1 Must Do, 5 Reductions (1M5R)”, which promotes six core principles: 1 Must Do = Use certified seed; 5 Reductions = Reduce seed rate, fertilizer use, pesticide use, water use and postharvest losses. In addition, a model, “Small Farmers, Large Field,” has been introduced to improve land use efficiency and productivity of rice production in the MRD and elsewhere in Asia^14–16^. The 1M5R approach applied in Vietnam increases nitrogen, water and pesticide use efficiency without compromising productivity and profitability^7,8^. A water-saving technology called “Alternate Wetting and Drying” has been also applied on a large scale in the MRD^4,17,18^. Such intermittent irrigation can reduce GHGEs by 40–50% compared to a continuously flooded rice production system^19–21^.

Crop establishment, which often receives insufficient attention, is one of the major rice production operations that should be considered when promoting sustainable rice production practices. Direct seeding (DSR) (e.g., broadcasting, drum-seeding, blower seeding), manual transplanting and mechanized transplanting are currently the common practices used in irrigated rice production^7,22–24^. DSR integrated with water-saving management has been reported as an advanced practice in terms of productivity, labor saving and water-use efficiency^25–28^. Mechanized transplanting has demonstrated advantages for irrigated lowland rice with yields reported to be 7% higher compared to manual transplanting, as well as having lower production costs^29,30^. Mechanized transplanting is currently at an early stage of adoption in the MRD^31^. There are available options for crop establishment, but a major research gap is quantitative data on the best approach in terms of sustainability. This study compared four crop establishment options: manual broadcasting (BroadC), blower seeding (BlowS), drum seeding (DrumS), and mechanized transplanting (MecT). The performance of these options was quantified for irrigated rice based on performance indicators for sustainable production. These indicators include grain yield, energy efficiency, GHG emissions, labor input, and net profit. BroadC is currently the common practice in the Mekong delta^8^. We hypothesize that MecT, although requiring a high upfront cost, will perform as well or better than BroadC and the other practices when assessed against performance indicators for sustainable rice production.

## Materials and methods

All methods included in the research, such as the experimental design, measurement of planting uniformity, yield, and sustainable performance indicators, are under the guidelines of the International Rice Research Institute (IRRI) or global standards, which are indicated in the specific sections and parameters below. The manuscript was internally reviewed and approved by IRRI.

### Site and crop descriptions, experimental design and water management

The experiment was conducted in Trung-Thanh Village, Co Do District, Can Tho, Vietnam (10.178103°N latitude; 105.524434°E longitude), across two consecutive rice-cropping seasons. These were the Winter-Spring season (WS), or dry season, from 8 November 2018 (sowing) to 14 February 2019 (harvest); and the Summer-Autumn season (SA), or early wet season, from 1 March 2019 (sowing) to 28 May 2019 (harvest). Rice varieties used were Dai-Thom-8 and OM5451 for the WS and SA seasons, respectively. The use of these plants complies with the national guidelines of Vietnam^32^. At the start of the WS season, fields were drained of floodwater, whereas, at the start of the SA season, irrigation water was required during land preparation before the onset of the monsoon rains. The mean farm size in the study area was 2.1 ± 0.1 ha, with an acid sulphate clay soil type^8^. The predominant crop establishment method was wet direct-seeding with broadcast pregerminated seed and the majority of farmers used four-wheel tractors for land preparation and combine harvesters for harvesting^8^.

**Figure 1:**
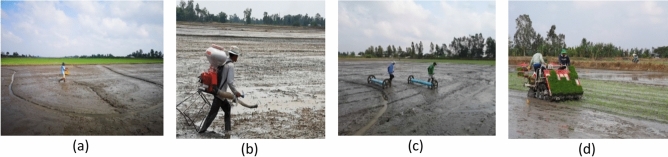
Four crop establishment practices showing (**a**) manual broadcasting (BroadC), (**b**) blower seeding (BlowS), (**c**) drum seeding (DrumS) and (**d**) mechanical transplanting (MecT). The people with their images included in Fig. 1 have consented to publish the paper as online open-access material.

**Figure 2:**
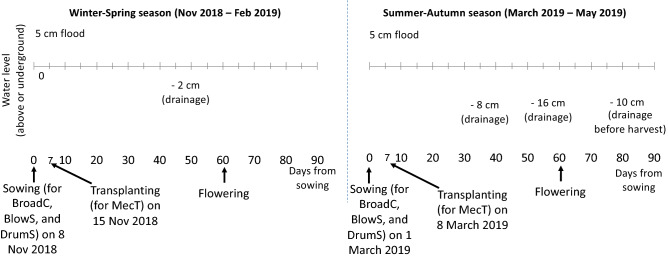
Water level (above- or under-ground) during the SA and WS cropping seasons.

**Figure 3:**
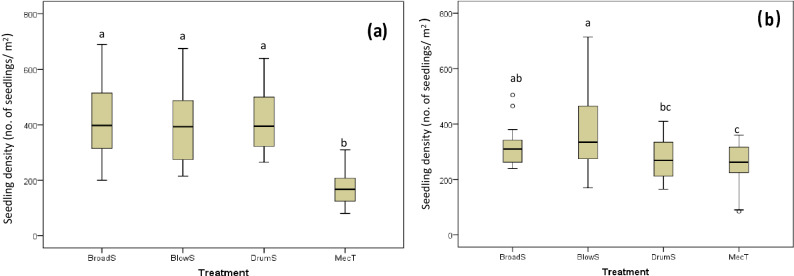
Seedling density (no. of seedlings m^-2^) 7 days after sowing/transplanting of the four different crop establishment treatments in Can Tho, Vietnam, during the Winter-Spring (**A**) and Summer-Autumn (**B**) seasons. Box plots with the same letters are not significantly different at the 0.05 level of significance following pairwise comparisons.

**Figure 4:**
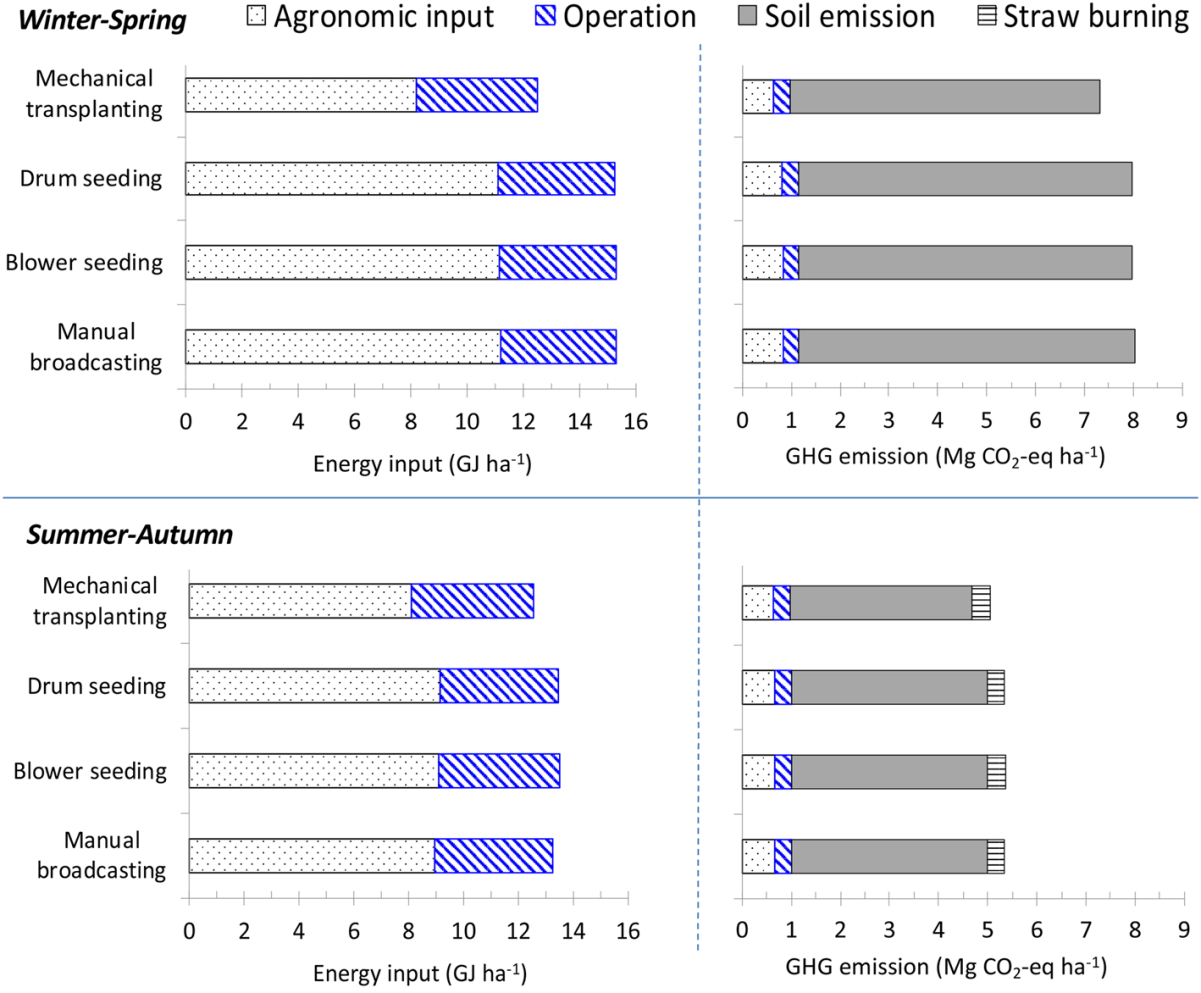
Energy input, GHGEs and production costs for rice production applied with 1M5R under different crop establishment options in Can Tho, Vietnam.

The four crop establishment methods were considered as separate treatments and implemented in a randomized complete block design (Table 1). The four treatments were: (1) BroadC (Fig. 1a), (2) BlowS using the Kasei 3WF-3A-26L machine (Fig. 1b), (3) DrumS using the Hoang-Thang drum seeder (Fig. 1c), and (4) MecT using the Yanmar VP7D25 transplanter (Fig. 1d). The four different farmers’ fields were considered as blocks or replicates and the four crop establishment methods were applied in each field (block). The field area of each treatment ranged from 3,000 to 4,000 m^2^. Different fields were used each season. Irrigation and drainage were applied similarly across the four treatments, but were different for the WS and SA seasons depending on the weather and flood conditions at the research site (Fig. 2). Growing time of the rice was 90 and 83 days for DSR and MecT, respectively. However, MecT required the seedlings to be prepared 12 days prior to crop establishment.

**Table 1:** Distribution of the treatments and replications in the experiment plots.

Farmer 1	Farmer 2	Farmer 3	Farmer 4
BroadC-1	BlowS-2	BlowS-3	MecT-4
BlowS-1	MecT-2	MecT-3	DrumS-4
DrumS-1	BroadC-2	DrumS-3	BroadC-4
MecT-1	DrumS-2	BroadC-3	BlowS-4

BroadC = manual broadcasting, BlowS = blower-seeding, DrumS = drum-seeding, MecT = mechanized transplanting; the numbers associated with the treatments in the Table (i.e. 1, 2, 3, and 4) represent for the blocks or farmers, correspondingly.

Land preparation, fertilizer and pesticide application, and harvesting operations, were the same for all treatments. Land preparation included plowing using locally fabricated rotavators and puddling with wet leveling. Fertilizer and pesticides were applied using Knapsack-blowers and -sprayers, respectively, combined in the Kasei 3WF-3A-26L machine. Harvesting in all treatment plots was done using combine harvesters (Kubota DC-70). Straw after harvest was generally incorporated at about 25–30 days before land preparation for the WS, while it is burned at about 12–15 days before land preparation for the SA. We applied 1M5R based on the criteria described in Table 2. We were not prescriptive for herbicide application. For all treatments, farmers applied herbicide based on their experience. Sofit-300EC (480 ml ha^-1^) and Cantamil-550EC (500 ml ha^-1^) were the two common herbicides used.

**Table 2:** Specifications of best practices for irrigated rice production (1 Must (certified seed) and 5 Reductions (reduced rates of seed, fertilizer, pesticides and water; reduced post-harvest losses) applied in the field trial at Trung-Thanh Village, Co Do District, Can Tho, for both seasons. (Max. = maximum).

Criteria*	Requirements	
Seed rate	≤ 120 kg ha^-1^	Certified seed
Nitrogen	≤ 100 kg ha^-1^	Applied with at least three splits
Insecticides	Max. 1 product application	No application within 40 days after sowing
Fungicides	Max. 2 product applications	No application after the flowering crop stage
Water management	Dry fields during the cultivation following AWD technique	
Harvesting	Combine harvester	Harvest when 80–85% of the grains per panicle are straw or yellow-colored

*Postharvest processes were excluded for analyses of the findings in this study.

### Measuring planting uniformity

To measure the planting uniformity, five 40- × 50-cm quadrants (randomly placed in a cross diagonal transect) were sampled in each treatment plot 7 days after sowing or transplanting. Seedling density was assessed by counting the number of seedlings in each quadrant divided by the quadrant area. The standard deviation (SD) was then used to compare the variation in seedling density from the mean across all replicate plots.

### Quantification of grain yield

Grain yield was determined by the crop-cut method from each experimental plot. In the WS season, the samples for the crop cut were taken from two 5-m^2^ (2.5 × 2.0 m) quadrants, which were located 5 m from the center of each plot along a cross diagonal transect. In the SA season, the same sampling procedure was applied with one more sampling at the center of each plot (total of three samples for each plot). The threshed paddy grains were cleaned (unfilled spikelets removed), weighed and recorded as fresh weight. The moisture content (MC) of the grain samples was determined using a grinding-type moisture meter (Kett®, product code: F511), which was precalibrated using the oven method^33^. The grain yield was calculated at 14% MC.

### Analysis of energy efficiency and indirect GHGEs

Energy efficency (GJ ha^-1^) was analyzed based on the net differences between the outputs and inputs of rice production—Eq. 1 (Eq. 1):

1 $${\text{NEV}} = {\text{E}}_{{{\text{out}}}} {-}{\text{ E}}_{{{\text{in}}}} \left( {{\text{GJ ha}}^{{ - {1}}} } \right)$$

where NEV is the net energy value for energy efficiency; E_out_ is the output energy value only accounting for the harvested grains but not including rice straw because this residue was incorporated before WS and burned before SA in this research; E_in_ is the input energy value accounting for mechanized operations including machine production and fuel consumption, labor and agronomic inputs such as seeds, fertilizer and pesticide. The conversion factors reported in Ecoinvent (2019)^34^ were used to estimate the energy of the related materials and processes (Table 3). In addition, the energy conversion factor for machine production was calculated through fuel consumption at 15 MJ L^-1 (35,36)^. Fertilizer inputs, such as nitrogen (N), phosphorus (P_2_O_5_) and potassium (K_2_O) were calculated based on the chemical content of N, P_2_O_5_ and K_2_O, such as urea (46–0-0) and DAP (18–46-0). Pesticide and herbicide inputs were converted based on the content of active ingredients and the conversion weight of the applied pesticides. Manpower was calculated based on the metabolic equivalent of task (MET), which is the ratio of human metabolic rate when performing an activity to the metabolic rate at rest, and on a labor energy conversion factor^37^, with the assumption that the mean weight of a Vietnamese is 55 kg.

**Table 3:** Energy and GHGE conversion factors used for calculating the relative energy efficiency of the four crop establishment methods from crop establishment to harvest.

Parameters	Energy			GHGE		
	Unit	Value	Sources	Unit	Value	Sources
Land use	MJ ha^-1^	0.0024	^34,41^	See details under “Soil emissions”		
Seeds	MJ kg^-1^	30.1	^34,41^	kg CO_2_-eq kg^-1^	1.12	^34,41,46^
Grain	MJ kg^-1^	15.2	^34,42^			
Diesel consumption	MJ L^-1^	44.8	^34,35,41^	kg CO_2_-eq MJ^-1^	0.08	^34,41,46^
Gasoline consumption	MJ L^-1^	39.1	^34,35,41^	kg CO_2_-eq MJ^-1^	0.08	^34,41,46^
Electric power	MJ kWh^-1^	3.6	^34,35,41^	kg CO_2_-eq kWh^-1^	0.564	^34,41^
Machine production	MJ L^-1^	15.6	^35,36^			
N	MJ kg^-1^	58.7	^34,41,43^	kg CO_2_-eq kg^-1^	5.68	^34,41,46^
P_2_O_5_	MJ kg^-1^	17.1	^34,41,43^	kg CO_2_-eq kg^-1^	1.09	^34,41,46^
K_2_O	MJ kg^-1^	8.83	^34,41,43^	kg CO_2_-eq kg^-1^	0.52	^34,41,46^
Herbicide	MJ kg^-1^	354	^34,41,44^	kg CO_2_-eq kg^-1^	23.3	^34,41,46^
Pesticide	MJ kg^-1^	182	^34,41,43^	kgCO_2_-eq kg^-1^	10.4	^34,41,46^
Driving 4WT and combine harvesters	MJ h^-1^	0.44	^37,45^			
Manual labor	MJ h^-1^	0.89	^37,45^			
**Soil emission:**						
EF_default_ of CH_4_ in WS				kg ha^-1^ day^-1^	1.7	^47^
EF_default_ of CH_4_ in SA				kg ha^-1^ day^-1^	2.8	^47^
SF_pre_ for pre-season soil management					1	^13^
SF_water_ for single drainage					0.71	^38^
SF_water_ for multiple drainage					0.55	^38^
SF_N_ for Nitrogen use				% N applied	0–1	^38^
CF_incorporation_					1	^13^
CH_4_ from burning straw				kg Mg^-1^	4.51	^40^
N_2_O from burning straw				kg Mg^-1^	0.069	^40^

GHGE (kg CO_2_-eq ha^-1^) is calculated based on Eq. (2), that accounts for the production of agronomic inputs including seeds, fertilizer and pesticide (GHG_agro-input_); mechanized operations (GHG_operation)_, soil emissions (GHG_soil_) and rice straw management (GHG_ricestraw_).

2 $${\text{GHGE }} = {\text{ GHG}}_{{{\text{agro}} - {\text{inputs}}}} + {\text{ GHG}}_{{{\text{operation}}}} + {\text{ GHG}}_{{{\text{soil}}}} + {\text{ GHG}}_{{{\text{ricestraw}}}} ({\text{kgCO}}_{{2}} - {\text{eqha}}^{{ - {1}}} {\text{season}}^{{ - {1}}} )$$

The GHG conversion factors for agronomic inputs and mechanized operation are shown in Table 3. GHG_soil_ is calculated based on Eq. (3)^38^, accounting for CH_4_ and N_2_O emissions. The CH_4_ emission is affected by water management, pre-season soil management and rice straw incorporation; while the N_2_O emission is affected by N use for rice cultivation^38^.

3 $$GHG_{soil} = \, Time_{grow} *28*EF_{default} * \, SF_{water} * \, SF_{pre} *SF_{ricestraw} + \, 265*EF_{1FR} *F_{fertilizer} (kgCO_{2} - eq \, ha^{ - 1} season^{ - 1} )$$

where Time_grow_ is the rice-growing period; 28 and 265 are the Global Warming Potentials of CH_4_ and N2O, respectively, for conversion to CO_2_-eq^38^; EF_default_, SF_water_ and SF_pre_, are the CH_4_ emission and scaling factors of water management and pre-season soil management, respectively; and SF_ricestraw_ is the scale factor for rice straw management. EF_1FR_ is the N_2_O emission factor in flooded rice systems and fertilizer amount of applied N, calculated based on Eq. (4)^38^. Water management was considered as single- and multiple-drainage scenarios during the WS and SA, respectively (Fig. 2). Total growing time of the direct-seeded rice was 90 days while that of transplanted rice in the field was 83 days. The seedling preparation time of 12 days was accounted for in the transplanted rice scenario. However, the land area used for seedling is only 1:100 for growing compared with the common practice in Vietnam, which was observed to be the case in this study. The emission and scaling factors are shown in Table 3.

4 $${\text{SF}}_{{{\text{ricestraw}}}} = (1 + {\text{R}}_{{{\text{straw}}}} *{\text{CF}}_{{{\text{straw}}}} )^{0.59}$$

where R_straw_ is the incorporation rate of rice straw (dry matter, t ha^-1^) and CF_straw_ is the conversion factor of rice straw depending on time of incorporation before the crop establishment. Yield of straw only accounted for top parts of rice plant harvested is 50% of rice yield^39^. This factor is only applied for the straw incorporation scenario of WS but not for the burning scenario of SA. On the other hand, GHG emission from straw burning is taken into account through the last component (GHG_ricestraw_) in Eq. (2) which is reported in Romasanta (2017)^40^.

### Computation of sustainability performance indicators

The Sustainable Rice Platform (SRP) has developed 12 sustainability performance indicators for rice production (SRP, 2019). We computed the seven agronomic indicators: productivity (grain yield), nitrogen-use efficiency (NUE), phosphorous-use efficiency (PUE), biodiversity (pesticide use), labor productivity, profitability (net profit) and GHGE as defined by SRP version 2^13^. In addition, we included potassium-use efficiency (KUE) due to its importance in rice productivity. Farmers were asked to record input and economic data in diaries, which were checked and collected by project staff every 3–4 weeks. To compute phosphorus (P) and potassium (K) application rates, the amounts of P_2_O_5_ and K_2_O for each fertilizer application were determined and multiplied by a factor of 0.4364 and 0.8302, respectively, to convert them into the elemental form^13^. To compute for NUE, PUE and KUE, the total grain yield harvested was divided by the elemental N, P or K rate applied and was expressed in terms of kg grain kg^-1^ elemental N, P or K. To compare pesticide practices among treatments, we reported the total frequency of application of formulated pesticide products. To compute for labor productivity, both hired and owned (family) male and female laborers were considered and the number of labor days per season (for all activities from land preparation until harvest, including regular field visits by farmers) were estimated by dividing the total labor cost per season by the average daily wage rate (VND 200,000 day^-1^, collected during this research) at the time taken across all activities. The result was then divided by the grain yield as determined from crop cuts.

Net income was calculated by deducting the total production cost from the gross income obtained from grain yield. Production cost consisted of: (1) land use; (2) service costs of mechanized operations such as land preparation, mechanical transplanting, fertilizer and pesticide applications and combine harvesting; (3) agronomic inputs including seeds, fertilizer and pesticide; and, (4) labor. Gross income consisted of the income from the total fresh harvested grain sold at the field. Costs of inputs and price of paddy are shown in Table 4.

**Table 4:** Cost of inputs and price of paddy.

Inputs	Unit	Value
Land use	$US ha^-1^ year^-1^	2000
Water pumping for WS	$US ha^-1^season^-1^	23
Water pumping for SA	$US ha^-1^season^-1^	34
Seed	$US kg^-1^	5.2
Urea 46-0-0	$US kg^-1^	58.7
TSP 18-46-0	$US kg^-1^	0.6
MOP 0-0-60	$US kg^-1^	0.4
NPK 16-16-16	$US kg^-1^	0.6
NPK 16–16-8	$US kg^-1^	0.5
Herbicide	$US L^-1^	4.8
Molluscicide	$US L^-1^	6.1
Fungicide/Insecticide	$US kg^-1^	12.3
Fungicide/Insecticide	$US L^-1^	11.0
Land preparation	$US ha^-1^	94.1
Manual broadcast-seeding	$US ha^-1^	26.0
Blower seeding	$US ha^-1^	26.0
Drum seeding	$US ha^-1^	26.0
Mechanized transplanting	$US ha^-1^	220.0
Crop care	$US ha^-1^	56.5
Harvesting	$US ha^-1^	90.3

### Statistical analysis and software

SPSS software and Analysis of Variance (ANOVA) were used to evaluate the effects of the contrasting crop establishment-based scenarios on the measured production and environmental parameters using a Least Significant Difference (LSD) at *α* = *0.05* to compare the mean values. Seedling density was analyzed using log transformation due to non-normally distributed residuals. Energy balance analysis was based on the Cumulative Energy Demand 1.09 method by SIMAPRO (2019)^41^ and CO_2_-equivalent analysis was based on the GWP-100a of IPCC (2013)^46^.

## Results

### Planting uniformity

During the WS and SA seasons, there was a significant difference in the seedling density among treatments (*F*_3,73_ = 39.050, *P* < 0.001; *F*_3,73_ = 4.984, *P* = 0.003, respectively). During the WS season, the seedling density for MecT was significantly lower than those of the other crop establishment methods (*P* < 0.05; Fig. 3). In addition, the variation in seedling density (or planting uniformity) for MecT was substantially lower (SD = 54.1) than BlowS (SD = 130.0), BroadC (SD = 137.8) and DrumS (SD = 104.9). During the SA season, there was no significant difference in the seedling density between Drum S and MecT. However, the seedling density for BlowS was significantly higher than DrumS and MecT. During this season, BlowS also had the largest variation in seedling density (SD = 146.9), and therefore had the lowest planting uniformity, compared to BroadC (SD = 70.6), DrumS (SD = 75.2) and Mec T (SD = 79.2). The average seedling density of MecT in the WS was lower than in the SA; while that of other treatments in the WS was lower than in the SA. The differences were mainly caused by the different operational performances during crop establishment.

### Energy input, GHGE, and production cost

Figure 4 shows the energy input, GHGEs and production costs for rice production among the different crop establishment options applied with 1M5R. The MecT had additional fuel consumption and machine production energy use than other direct seeding treatments but had lower agronomic inputs, particularly the seed rate, which was 50–60 kg ha^-1^, as compared with 100–120 kg ha^-1^ for the other DSR treatments. MecT also had lower in-field growing time than direct seeding by about 10%. These together led to lower total energy input and GHGEs of MecT than for direct seeding. Total energy input was 12.5–15.3 GJ ha^-1^ and 12.6–13.5 GJ ha^-1^, during the WS and SA seasons, respectively, consisting of 65–73% from agronomic inputs and the rest from operations. GHGEs during WS were 7.31–8.03 Mg CO_2_-eq ha^-1^, higher by 40% than that during SA; mainly caused by the difference of rice straw management (incorporation before the WS and burning before the SA) and water management (one drainageof rice fields during the WS and two drainages during the SA). Of the total GHGEs during the WS and SA, respectively, 86 and 70% were from soil emissions; 10 and 14% were from agronomic inputs; 4 and 8% were from mechanized operations and 8% was from straw burning (for SA).

### Sustainability performance indicators

**Figure 5:**
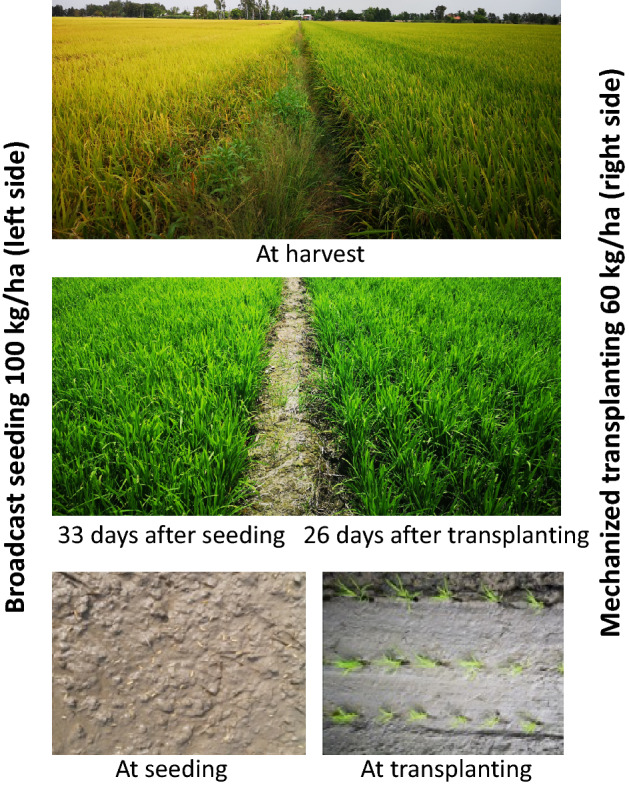
Comparisons in experimental fields of rice sown using a blower seeder (broadcast seeding, on left) or mechanized transplanting (on the right).

Table 5 shows the sustainability performance indicators of crop production across the four field trial treatments in the WS and SA seasons. There was no significant difference in N-P-K use efficiency (n = 4, *P* > 0.05). However, the farmers used less fertilizer for the MecT in the WS because of better rice plant growth and leaf color. As a consequence, MecT also had better mean energy efficiency. MecT had significantly lower pesticide use in the WS season. There was a significant difference in the number of pesticide applications between treatments (*F*_9,3_ = 5.121, *P* = 0.024), with the lowest number applied in the MecT treatment (*P* < *0.005*). MecT required less pesticides because of increased rice plant vigor and lower plant density (Fig. 5). During the SA season, farmers applied less fertilizer and pesticide than in the WS season, with no significant differences across treatments. However, there was a significant difference in labor productivity among treatments (*F*_9,3_ = 5.498, *P* < 0.001). Labor productivity was significantly lower in the MecT plots compared to the other treatments (*P* < 0.05).

**Table 5:** Sustainability performance indicators (mean values followed by standard error in parentheses) of crop production across the four field trial treatments in the WS and SA seasons in Can Tho, Vietnam.

	Manual broadcasting		Blower seeding		Drum seeding		Mechanical transplanting	
	(n = 4)		(n = 4)		(n = 4)		(n = 4)	
**Winter-Spring**								
Nitrogen-use efficiency (grain kg N kg^-1^)	80.5	(14.56)	78.9	(14.55)	78.5	(11.80)	101.8	(17.34)
Phosphorus-use efficiency (grain kg P kg^-1^)	322.4	(55.59)	312.8	(46.65)	315.2	(41.77)	358.6	(16.81)
Potassium-use efficiency (grain kg K kg^-1^)	185.3	(24.43)	180.3	(19.92)	181.0	(14.46)	248.8	(30.00)
No. of pesticide applications	9.8	(1.44)^a^	9.8	(1.44)^a^	9.8	(1.44)^a^	6.5	(0.87)^b^
Labor productivity (kg days^-1^) – based on total labor cost	264.3	(22.29)	262.4	(26.80)	269.4	(21.98)	254.9	(22.88)
Grain yield (t ha^-1^)	7.5	(0.44)	7.4	(0.48)	7.4	(0.27)	7.5	(0.23)
Energy efficiency (GJ ha^-1^)	98.61	–	96.74	–	97.54	–	101.64	
GHGEs (kg CO_2-_eq ha^-1^)	8,025	–	7,988-	–	7,976	–	7307	
Net income (USD ha^-1^)	1,014	(96)	999	(101)	1,017	(56)	1069.8	(60)
**Summer-Autumn**								
Nitrogen-use efficiency (grain kg N kg^-1^)	83.9	(6.28)	83.2	(5.64)	77.6	(6.46)	83.2	(7.76)
Phosphorus-use efficiency (grain kg P kg^-1^)	336.4	(29.27)	333.4	(27.15)	310.2	(25.42)	341.3	(39.73)
Potassium-use efficiency (grain kg K kg^-1^)	174.9	(7.49)	173.7	(7.66)	161.9	(9.16)	177.1	(14.09)
No. of pesticide applications	3.5	(0.29)	3.5	(0.29)	4.0	(0.71)	3.5	(0.65)
Labor productivity (kg days^-1^)—based on total labor cost	255.7	(8.86)^a^	241.9	(17.29)^a^	228.4	(17.99)^a^	136.9	(8.09) ^b^
Grain yield (t ha^-1^)	6.7	(0.10)	6.6	(0.21)	6.2	(0.36)	6.8	(0.27)
Energy efficiency (GJ ha^-1^)	87.86	–	87.04	–	80.45	–	90.60	
GHGEs (kg CO_2-_eq ha^-1^)	4,984	–	4,991	–	4995	–	4,679	
Net income (USD ha^-1^)	769	(25)	749	(54)	663	(663)	678	(92)

Within a particular row, numbers followed by different letters are significantly different by least significant difference at α = 0.05.

## Discussion

The use of 1M5R practices following mechanized crop establishment produced similar yields to the other three crop establishment methods. More importantly, MecT provided significant economic and environmental benefits to smallholder farmers in the MRD because of reduced input use. The findings support what we hypothesized. Although this study was conducted under the conditions of irrigated rice for a specific site in Can Tho, we suggest that the findings are representative of most of the lowland rice production in the MRD with alluvial soil.

Rice production in the MRD has high yield (about 6–7 Mg ha^-1^) compared with most other countries in Southeast Asia^1^, but the net income of farmers is low mainly due to overuse of seed, fertilizer and pesticides^7,8^. In contrast, we demonstrate in this study that the application of 1M5R and mechanized transplanting, using lower agronomic inputs without reducing yield, generated a net profit of 600–1,000 $US ha^-1^ season^-1^ or about 1,800–3,000 $US ha^-1^ for three crops per year. This equals 0.2–0.3 $US kg^-1^ paddy, which was 7–20% higher than FP and 20–40% higher than that reported for conventional farmers in Devkota et al. (2019)^7^ in the same region of the MRD.

Our findings do not clearly demonstrate the influence of the crop establishment method on energy efficiency and GHGEs, but revealed that mechanized transplanting did not increase energy input and GHGEs (based on life-cycle assessment)^48^. A number of studies highlight the advantages of direct seeding practices under wet-tillage condition in terms on yield, water use effciency and labor^27,28^. However, in the previous studies, the comparisons were compared to manual transplanting and not to mechanized transplanting, a technology that has been significantly improved recently. The current study illustrates that mechanized transplanting reduced seed rate by 40% compared to three other direct seeding options. Moreover, the reduction of sowing density, as well as the planting of 12-day old seedlings, led to reduced fertilizer and pesticide use. The latter suggests that mechanical transplanting reduces weeds, pest and disease pressure in comparison with wet direct-seeding. This is likely to be due to a number of factors, such as reduced exposure of seeds and young seedlings in fields to birds, snails and rats; a competitive advantage of rice seedlings over weeds after transplanting, and lower plant densities that lead to more ventilation and lower humidity.

Herbicide application was based on conventional farmer practice and was the same for all treatments. We contend that mechanized transplanting would reduce or avoid herbicide application through enabling better vigor of rice seedlings after the field had been mechanically cultivated to manage weeds. The benefits of manual transplanting over wet direct seeding in relation to weeds, pests and diseases are well documented^49–51^. We argue that our results highlight similar benefits for MecT. The density of seed-trays and age of seedlings when transplanting also are critically important factors to consider when transplanting in snail-infested regions^49^. Through reduced seed rates, mechanized transplanting also reduced the risk of the lodging of rice plants^50^. Reduced lodging was observed in the current study and thus reduced postharvest losses due to unfavorable operating conditions of combine harvesters when the crop is lodged. The grain quality of lodged rice also is significantly reduced because of increased moisture content of the grain and mud contaminating the grain.

Energy efficiency, GHGEs and net profit are commonly used as environmental and economic indicators of crop production^13,48^. Energy efficiency, which is the net energy difference between outputs and inputs of rice production, could vary depending on many factors including site-specific management of water, nutrients, pests and crop residues. Previous studies report a wide range for the estimate of net energy value for irrigated rice: 13.7 MJ kg^-1^ rice produced in Ecoinvent (2019)^34^; 11.3–12.3 MJ kg^−1^ for rice in the Philippines in Quilty et al. (2014)^45^; and 10–28 MJ kg^−1^ for production in the Philippines with different rice straw management practices^36^. The estimates of total input energy of rice production in the current study are similar to those reported for irrigated rice production in Southeast Asian^36,45,52^. The energy efficiency value in the current study (11–14 MJ kg^-1^) was similar with that reported in Ecoinvent (2019)^34^ and was higher by 10% than that reported in Quilty et al. (2014)^45^, which is likely because of higher grain yield in the MRD compared to that produced in the Philippines.

In this study, soil emission levels were calculated based on the conversion factors reported in IPCC (2019)^38^ using research scenarios with similar specific water and rice straw management and fertilizer application. Total GHGEs of the research scenarios were 1.05 and 0.65 kg CO_2_-eq kg^-1^ paddy during WS and SA, respectively. GHGEs during WS was higher by 40% that of SA and that reported in Vo et al. (2017)^53^ because of the additional emissions from rice straw incorporation.

## Conclusions

The research provided field-trial evidence from studies within smallholder farmers’ fields of the benefits of mechanized transplanted rice compared to direct seeded rice in the MRD. Across the four treatments, the rice yield ranged from 7.3 to 7.5 Mg ha^−1^ and 6.2 to 6.8 Mg ha^−1^ in the WS and SA seasons, respectively. In comparison with direct seeding methods, the mechanized transplanting practice decreased the seed rate by 40% and reduced pesticide applications by 30–40% in the main crop season (WS) of Vietnam. Despite mechanized transplanting required higher inputs for machine production (depreciation) and fuel consumption, its net energy balance, net income and GHGEs were at a similar level as the other non-mechanized planting practices. Thus, MecT in combination with 1M5R is a technology package that should be promoted to improve the economic and environmental sustainability of rice cultivation in the MRD.

## List of Symbols

$US: US dollar
1M5R: 1 Must Do, 5 Reductions
4WT: Four-wheel tractor
AWD: Alternate Wetting and Drying
BlowS: Blower-seeding
BroadC: Manual broadcasting
CO_2_-eq: Carbon dioxide equivalent
CRA: Climate resilience agriculture
DrumS: Drum-seeding
DSR: Direct seeding
G.A.P: Good Agricultural Practice
GHGEs: Greenhouse gas emissions
h: Hour
ha: Hectare
kg: Kilogram
kWh: Kilowatt*hour
K_2_O: Potassium
KUE: Potasium-use efficiency (grain kg potasium kg^-1^)
L: Liter
LCA: Life Cycle Assessment
MC: Moisture content in wet basis
MET: Metabolic equivalent of task
MecT: Mechanized transplanting
Mg: Megagram
MJ: Megajoules
MRD: Mekong River Delta of Vietnam
N: Nitrogen
NUE: Nitrogen-use efficiency (grain kg nitrogen kg^-1^)
P_2_O_5_: Phosphorus pentoxide
PUE: Phosphorus-use efficiency (grain kg phosphorus kg^-1^)
SA: Summer-Autumn
SFLF: Small Farmers, Large Field model
SRP: Sustainable Rice Platform
WS: Winter-Spring
